# The Role of Pseudo-Orthocaspase (SyOC) of *Synechocystis* sp. PCC 6803 in Attenuating the Effect of Oxidative Stress

**DOI:** 10.3389/fmicb.2021.634366

**Published:** 2021-02-04

**Authors:** Saul Lema A, Marina Klemenčič, Franziska Völlmy, Maarten Altelaar, Christiane Funk

**Affiliations:** ^1^Department of Chemistry, Umeå University, Umeå, Sweden; ^2^Biomolecular Mass Spectrometry and Proteomics, Bijvoet Center for Biomolecular Research, Utrecht Institute for Pharmaceutical Sciences, University of Utrecht, Utrecht, Netherlands; ^3^Netherlands Proteomics Centre, Utrecht, Netherlands

**Keywords:** pseudo-enzyme, orthocaspase, *Synechocystis* sp. PCC6803, proteomics, programmed cell death

## Abstract

Caspases are proteases, best known for their involvement in the execution of apoptosis—a subtype of programmed cell death, which occurs only in animals. These proteases are composed of two structural building blocks: a proteolytically active p20 domain and a regulatory p10 domain. Although structural homologs appear in representatives of all other organisms, their functional homology, i.e., cell death depending on their proteolytical activity, is still much disputed. Additionally, pseudo-caspases and pseudo-metacaspases, in which the catalytic histidine-cysteine dyad is substituted with non-proteolytic amino acid residues, were shown to be involved in cell death programs. Here, we present the involvement of a pseudo-orthocaspase (SyOC), a prokaryotic caspase-homolog lacking the p10 domain, in oxidative stress in the model cyanobacterium *Synechocystis* sp. PCC 6803. To study the *in vivo* impact of this pseudo-protease during oxidative stress its gene expression during exposure to H_2_O_2_ was monitored by RT-qPCR. Furthermore, a knock-out mutant lacking the pseudo-orthocaspase gene was designed, and its survival and growth rates were compared to wild type cells as well as its proteome. Deletion of SyOC led to cells with a higher tolerance toward oxidative stress, suggesting that this protein may be involved in a pro-death pathway.

## Introduction

Cyanobacteria are essential players of the phytoplankton ecosystem; however, their uncontrolled growth can lead to harmful blooms and; therefore, can cause ecologic and economic damage (Wells et al., 2015). Understanding the fundamental mechanisms of Regulated Cell Death (RCD, reviewed in Galluzzi et al., 2018; Tang et al., 2019) in cyanobacteria; therefore, will be of highest relevance. By now, it is widely recognized that bacteria and other single-cell organisms execute programs, reminiscent of regulated cell death in response to environmental stimuli (Bayles, 2014; Peeters and de Jonge, 2018). These pathways also contribute to the mortality and maintenance of marine ecosystems by mediating the oceans’ phytoplankton blooms (Bidle, 2015, 2016). In metazoans, caspases are a well-studied family of evolutionary conserved cysteine-dependent aspartate-directed proteases recognized as primary regulators of apoptosis, and indispensable for its initiation and execution. Caspases are synthesized as zymogens (inactive forms) containing two subunits of ∼20 kDa (p20) and ∼ 10 kDa (p10), respectively. The presence of the histidine-cysteine (HC) dyad within the p20 domain is a requirement for their proteolytic activity (Riedl and Salvesen, 2007; Van Opdenbosch and Lamkanfi, 2019). Based on their structure and activation modes caspases are divided into two types: (a) initiator caspases, which are activated via dimerization of two p10 domains, and (b) executioner caspases, which need proteolytic separation of the large (p20) and small (p10) subunits. Initiator and executioner caspases cleave their substrates after aspartic acid residues (Riedl and Salvesen, 2007; Ramirez and Salvesen, 2018; Van Opdenbosch and Lamkanfi, 2019). Despite the unquestionable importance of these proteases in animals, caspases have not been identified in any other kingdom. A refined *in silico* analysis using the catalytic p20 domain as a query detected structurally highly homologous proteins in plants and fungi, which were termed metacaspases (Uren et al., 2000). Although metacaspases share similar structural components with caspases (they contain the p20 and p10 subunits), they lack their distinct aspartate substrate specificity. Instead, metacaspases cleave their substrates after lysine (K) or arginine (R) residues at the P1 position (Vercammen et al., 2004; Bozhkov et al., 2005; Watanabe and Lam, 2005; Tsiatsiani et al., 2011). Depending on their domain structure metacaspases are subdivided into type I and type II (Uren et al., 2000; Minina et al., 2017, 2020). Type I metacaspases, which are ubiquitous and can be found in plants, fungi and bacteria, can contain an N-terminal extension with proline-rich repeats and zinc-finger motifs (Dietrich et al., 1997; Uren et al., 2000). On the other hand, type II metacaspases are exclusively found in the green lineage and are classified by the presence of a prolonged (about 130 amino acid residues) interdomain region separating the p20 and p10 domains (Uren et al., 2000; Choi and Berges, 2013). Recently, genes encoding a third type of metacaspase have been identified, solely in algae that putatively arose from secondary endosymbiosis (Choi and Berges, 2013). These were termed type III metacaspases and had an unusual rearrangement of domains, with the p10-like domain at the N-terminus of the protein. Besides, in prokaryotes and some algae, caspase-homologs lacking the regulative p10 domain have been identified, which were termed metacaspase-like proteins in eukaryotes (Choi and Berges, 2013) and orthocaspases in prokaryotes (Klemencic et al., 2015; Klemencic and Funk, 2018). Strains belonging to α-proteobacteria, δ-proteobacteria, and cyanobacteria are particularly rich in the number of orthocaspases (Jiang et al., 2010; Asplund-Samuelsson et al., 2012). Cyanobacterial orthocaspases were believed to prefer caspase substrates (Berman-Frank et al., 2004; Bar-Zeev et al., 2013), however, *in vitro* prokaryotic orthocaspases do not recognize substrates with aspartic acid (D) residue at the P1 position, but instead prefer positively charged R or K residues (Klemencic et al., 2015; Spungin et al., 2019). Interestingly, many bacterial orthocaspases contain substitutions in the HC catalytic site; instead of the catalytic dyad, they display a highly variable pair of residues (Y-S, Y-N, Y-C, H-G,-C-Y, Y-R, Y-G, Y-Y, Y-H, Y-Q, or H-P), which presumably renders them proteolytically inactive (Klemencic et al., 2019; Bhattacharjee and Mishra, 2020). Analyzing cyanobacterial genomes, it was observed that all cyanobacteria containing orthocaspases contain a proteolytically inactive variant. These proteases were termed pseudo-orthocaspases. In unicellular cyanobacteria that only contain a single orthocaspase gene, it is always the pseudo-orthocaspase that is present (Klemencic et al., 2019).

Similar to multicellular organisms, RCD in cyanobacteria is a tightly genetically controlled mechanism orchestrated by a specialized molecular machinery (Zheng et al., 2013). Cyanobacterial RCD can be triggered by oxidative stress to contribute to the population’s survival (Blanco et al., 2005; Ross et al., 2006, 2019; Hu et al., 2011; Ding et al., 2012). The filamentous, diazotrophic cyanobacterium *Trichodesmium erythraeum* IMS101 is so far the only cyanobacterium, in which expression levels of the orthocaspases have been monitored during induced cell death. It encodes 12 orthocaspases, 9 true orthocaspases (TeMC1-TeMC9) containing the HC catalytic dyad, and 3 pseudo-orthocaspases (TeMC10-TeMC12) (Spungin et al., 2019). So far, only the true orthocaspases have captured the researchers’ attention (Bar-Zeev et al., 2013; Spungin et al., 2019). The complexity and high diversity of the prokaryotic p20-containing proteins and especially their possible role in stress response programs make research on cyanobacterial orthocaspases an exciting endeavor.

The unicellular cyanobacterium *Synechocystis* sp. PCC 6803 only contains a single orthocaspase (SyOC), which lacks the catalytic HC dyad and is presumably proteolytically inactive (Klemencic et al., 2019). Oxidative stress is known to have high impact on cyanobacterial growth (Latifi et al., 2009), and eukaryotic metacaspases are known to protect single cell organisms like yeast against it (Lefevre et al., 2012; Hill and Nystrom, 2015). In the present study, we investigated the role of the pseudo-orthocaspase during oxidative stress. A mutant depleted of SyOC was generated; its phenotype as well as its proteome were compared to wild type grown at normal conditions and during oxidative stress. Given that cells lacking SyOC had a better ability to tolerate oxidative stress suggests that the pseudo-orthocaspase found in *Synechocystis* sp. PCC 6803 may be involved in a pro-death pathway.

## Materials and Methods

### Cell Culture and Growth Conditions

The unicellular cyanobacterium *Synechocystis* sp. PCC 6803 (received from Vermaas lab, Arizona State University, United States) was maintained at 20 μmol photons m^–2^s^–1^ on plates containing BG-11 medium (Rippka et al., 1979), buffered with TES [N-tris(hydroxymethyl)-2-aminoethanesulfonic acid]-NaOH, pH 8.0, supplemented with 1.5% (w/v) Difco agar, 0.3% (w/v) sodium thiosulphate and appropriate antibiotics when required. Liquid cultures were grown photoautotrophically in BG-11 medium at 30°C, in an orbital shaker (120 rpm) under continuous illumination (30 μmol m^–2^s^–1^). All experiments were performed on cultures in their exponential growth phase (OD_730_ of 0.6–1), measured using a Jasco FP−6300 spectrofluorometer, Silver Spring, MD, United States).

### Deletion of the *syOC*

To knock-out the orthocaspase gene (*sll0148*), the coding sequence as well as the framing 500 bp up and down-stream (used as homologous recombination sites, HRS, Supplementary Figure 1A) were amplified from wild type (WT) genotype using the primers deltaSyOC_Eco_F and deltaSyOC_Eco_R (Supplementary Table 1). The amplified product was inserted into the cloning vector pJET1.2/blunt (Promega, Wisconsin, United States). The *apa*I and *sma*I restriction sites of *sll0148* were used to insert an antibiotic cassette conferring resistance to kanamycin (Km^*r*^) from pUC4K vector (Pharmacia Biotech, Sweden) (Supplementary Figure 1A). The pJET1.2/blunt vector containing HRS1_SyOC_HRS2 with the kanamycin resistance cassette was then used to naturally transform the *Synechocystis* sp. PCC 6803 WT strain. Briefly, cells were first grown in BG11 medium supplemented with 5 mM glucose until the OD reached 0.5. Cells were then collected by centrifugation and resuspended so that the final OD was >2. To 0.2 ml of cells, plasmid was added to the final concentration of 10 μg/ml. This mixture was incubated at 30°C for 3 h without shaking, followed by 3 h with shaking. Cells were then plated on a sterile filter laid on top of a BG11 plate supplemented with 5 mM glucose. The plate was left in the growth chamber for 1 day, then the filter was moved to a petri dish containing BG11 without glucose but supplemented with 5 μg/ml Km. Colonies appeared after approximately 14 days and were streaked on plates with continuously raising Km concentrations. Transformants were grown on BG11 plates containing increasing concentrations of Km (10–50 μg/ml) to allow segregation. Homozygosity of the mutants was confirmed using polymerase chain reaction (PCR).

### Abiotic Stress Treatment

Wild type *Synechocystis* sp. PCC 6803 and ΔOC mutant cells were exposed to oxidative stress, utilizing at least three biological replicates per treatment. An inoculum from the agar plate was allowed to grow in liquid culture for 3 days. The culture was then diluted with fresh media (150 ml) to an OD_730_ 0.1 and allowed to grow for 3 more days. At an OD_730_ of 0.6–0.8 the cells were subjected to oxidative stress via exposure to 0, 2, 3.5, 5, or 10 mM H_2_O_2_ (final concentration). Samples were collected after 0.5, 1, 3, and 6 h. Cells were harvested by centrifugation at 2,000 × g for 5 min. The supernatant was discarded, and the pellet was flash frozen in liquid nitrogen and stored at -80°C until further use. Samples for growth curve (OD_730_) assays were measured at 0, 1, 3, 6, 12, and 24 h.

### Photochemical Efficiency Measurements

A hand-held PAM fluorometer (AquaPen-C Ap-C 100, PSI, Drasov, Czech Republic) was used to measure the maximum quantum yield of Photosystem II (Φ or Fv/Fm) and monitor the photosynthetic performance of samples exposed to above listed H_2_O_2_ concentrations at 0, 1, 3, 6, 12, and 24 h. Measurements were performed after incubating the samples for 15 minutes in darkness.

### Quantification of Cyanobacterial Growth

Cyanobacterial growth was examined by plating 10-fold dilutions of the WT and ΔOC cultures previously grown in BG-11 liquid media (OD_730_ = 1) to the same OD, on BG-11 medium plates containing 0, 2, 3.5, 5, and 10 mM of H_2_O_2_ then incubated at 30°C for 3–4 days under continuous illumination (30 μmol m^–2^s^–1^). Colony Forming Units (CFUs) were counted by scanning the photo and using imagej for automated quantification. Cyanobacterial growth was calculated as CFUs/ml. Three biological replicates were used for growth quantification in each experiment. Plates were scanned using a scanner Epson perfection 3200 and quantified using Fiji software (Image J distribution) (Schindelin et al., 2012).

### RNA Extraction

RNA was extracted from at least three biological replicates of *Synechocystis* sp. PCC 6803 cells exposed to oxidative stress or grown at normal growth conditions as a control. RNA was isolated from 10 ml of cell culture in the early exponential phase using an RNAqueous^TM^. Total RNA Isolation Kit (Invitrogen, Thermo Fisher Scientific, Waltham, United States) according to the manufacturer’s instructions and subsequently treated with rDNAse I (Thermo Fisher Scientific, Wasltham, United States) to remove the remaining DNA. cDNA synthesis was performed after DNAse treatment using iScript cDNA synthesis kit (Bio-Rad, Life Science, Sweden) using random hexamer primers. After synthesis, the reaction mixture was diluted by a factor of 10 and used either as a template for quantitative reverse transcription real-time polymerase chain reaction (qRT-PCR) experiments. Quantity of isolated RNA was determined using a NanoDrop UV–Visible spectrophotometer (NanoDrop 2000 C, Thermo Fisher Scientific, Waltham, United States).

### RT-qPCR

The orthocaspase gene sequence was selected from the available genome of *Synechocystis* sp. PCC 6803^1^, and oligonucleotide primers used in this study were designed using Primer Blast software^2^. The analyses of gene expression were performed by real-time quantitative PCR (RT-qPCR), primer sequences are listed in Supplementary Table 1. As control genes, two housekeeping genes *rnpB* and *16S* were chosen (Pinto et al., 2012). The expression of all genes was analyzed using RT-qPCR with three technical replicates from three biological samples. RT-qPCR was performed in 10 μl reactions using SsoAdvanced^TM^ Universal SYBR Green Supermix (Bio-Rad, Life Science, Sweden) in a BioRad CFX 96 machine. After completing the cycle, melting curves were analyzed for all samples to confirm the specificity of amplification.

### Cell Counting and Viability Test

*Synechocystis* sp. PCC 6803 cell cultures were grown in the presence or absence of 2 or 3.5 mM H_2_O_2__;_ samples were collected at 6, 12, and 24 h and stained with SYTOX Green (Thermo Fisher Scientific, Waltham, United States) at a final concentration of 1 μM for 10 min under dark conditions (Schulze et al., 2011). Cells were examined and counted using a DMi8 inverted fluorescence microscope (Leica, Heidelberg, Germany), equipped with a DAPI/FITC/TEXAS RED filter. Images were acquired with a Leica DFC9000GT camera controlled by Leica Application Suite X software. At least five random fields from three biological replicates were used for viability calculations in each experiment and quantified by Fiji Image J distribution (Schindelin et al., 2012).

### Protein Purification and Digestion for Proteomic Analysis

*Synechocystis* sp. PCC 6803 WT and the ΔOC knock-out mutant line, previously grown in BG-11 medium until they reached an OD_730_ of 0.8, were treated with 3.5 mM H_2_O_2_ for 1 h. Then 30 ml of cell culture was harvested by centrifugation at 5,000 × g for 20 min at 4°C. The supernatant was discarded and the pellet was resuspended in 1 ml of lysis buffer (7 M Urea, 2 M thiourea, 4% CHAPS (w/v), 2% ASB-14 (w/v), 1% DM (w/v), 200 mM KCl, 100 mM of HEPES pH 7.0, 5 mM DTT) and complete^TM^ protease inhibitor cocktail (Merck, Darmstadt, Germany) (according the manufacturer’s recommendations). Then cells in lysis buffer were collected in MN Bead Tubes Type A (Macherey-Nagel, Dueren, Germany) and broken using a bead-beater in a cold (4°C) room. Cell debris and other insoluble proteins were removed by centrifugation at 21,000 × g for 30 min at 4°C. Finally, all samples were cleaned using a 0.2 μm non-pyrogenic sterile filter and collected in a new tube. The total protein concentration was determined using the BioRad RC DC protein assay kit (Bio-Rad, Hercules, CA, United States). Total extract was flash frozen in liquid nitrogen and stored at −80°C until further analysis.

Samples were processed using the S-trap micro spin column system (Protifi, Huntington, NY) according to the manufacturer’s guidelines, with the following changes. Lysis buffer was added to the samples in order to introduce SDC, and Tris was used in place of TEAB in all buffers. After elution from the S-trap spin columns, the samples were dried and resolubilized in 1% formic acid (FA) before mass spectrometric injection of a total of 1 μg.

### Mass Spectrometry: RP-NanoLC–MS/MS

Data were acquired using an Ultimate3000 system (Thermo Fisher Scientific) coupled to an Orbitrap Q Exactive HF-X mass spectrometer (Thermo Fisher Scientific). Peptides were first trapped (Acclaim PepMap100 C18, 5 μm, 100A) before being separated on an analytical column (Agilent Poroshell EC-C18, 2.7 μm, 50 cm × 75 μm). Trapping was performed for 2 min in solvent A (0.1 M FA in water), and the gradient was as follows: 9–13% solvent B (0.1 M FA in 80% ACN) in 3 min, 13-44% in 95 min, 44–95% in 3 min, and finally 100% for 4 min. The mass spectrometer was operated in data-dependent mode. Full-scan MS spectra from m/z 375–1,600 were acquired at a resolution of 60,000 at m/z 400 after accumulation to a target value of 3 × 106. Up to 15 most intense precursor ions were selected for fragmentation. HCD fragmentation was performed at a normalized collision energy of 27 after accumulation to a target value of 1 × 105. MS/MS was acquired at a resolution of 30,000.

### Data Analysis Using MaxQuant

Raw files were processed using MaxQuant (version 1.614.0). The database search was performed against the *Synechocystis* sp. PCC 6803 Uniprot database (UP000001425 3507) entries using Andromeda as a search engine. Cysteine carbamidomethylation was set as a fixed modification and methionine oxidation, protein N-term acetylation, and phosphorylation of serine, threonine, and tyrosine were set as variable modifications. Trypsin was specified as enzyme and up to two miss cleavages were allowed. Filtering was done at 1% false discovery rate (FDR) at the protein and peptide level. Label-free quantification (LFQ) was performed and quantified data were processed and analyzed using R and Perseus (Tyanova et al., 2016). Processed data can be found in Supplementary Table 2.

### Statistical Analysis

RNA expression was normalized to both housekeeping genes and further normalized against control expression of each treatment. One-way analysis of variance (ANOVA) was performed using GraphPad Prism version 8.0 (GraphPad Software Inc., La Jolla, CA, United States).

Following peptide identification by LC/MS and processing using MaxQuant, to provide robustness to the statistical analysis, non-valid detections were filtered. Only protein groups with at least two identified unique peptides over all runs were considered for further analysis. For quantification we combined related biological replicates to groups, which then were filtered using stringent confidence parameters: (i) proteins were filtered according to categorical columns (potential contaminants, reverse, and only identified by site); (ii) label-free values were transformed to logarithmic scale (Log_2_) and subtracted according to the median value of the columns to normalize the dataset; (iii) values were filtered based on a row with a minimum of 8 values out of 16 in total. Finally, we investigated only proteins found in at least one group in a minimum of 3 out of 4 biological replicates. To detect significant differences among conditions, we used Perseus software and performed a two-sample- test, using following settings: S_0_ = 0.5, both sides, permutation-based FDR = 0.01, 250 randomizations and a minimum of 3 values out 4 in both groups. The results of the test are summarized in Supplementary Tables 3, 4.

### Network Construction

To analyze the interactome resulting from the analysis, we analyzed the curated data set and performed a Hawaii plot (Supplementary Figure 2) using a network module (Rudolph and Cox, 2019) from Perseus software. We generated a network for each experimental condition selecting only enriched proteins (right side) with Perseus software as described in the user guide. The resulting networks were uploaded to Cytoscape (v. 3.8.2) and the nodes clustered using the Community cluster (GLay) algorithm from clusterMaker assuming that edges are undirected. To improve visualization, all the edges (number of handles: 3; spring constant: 0.003; compatibility threshold: 0.3; maximum iterations: 500) were bundled and used the corresponding information available from STRING regarding experimental evidence, databases, and coexpression analysis to establish putative interactions (edges).

### Data Availability

The mass spectrometry proteomics data have been deposited into the ProteomeXchange Consortium via the PRIDE (Vizcaino et al., 2016) partner repository^3^ with the dataset identifier PXD022955.

## Results

### Characterization of the Orthocaspase Knock-Out Mutant

**FIGURE 1:**
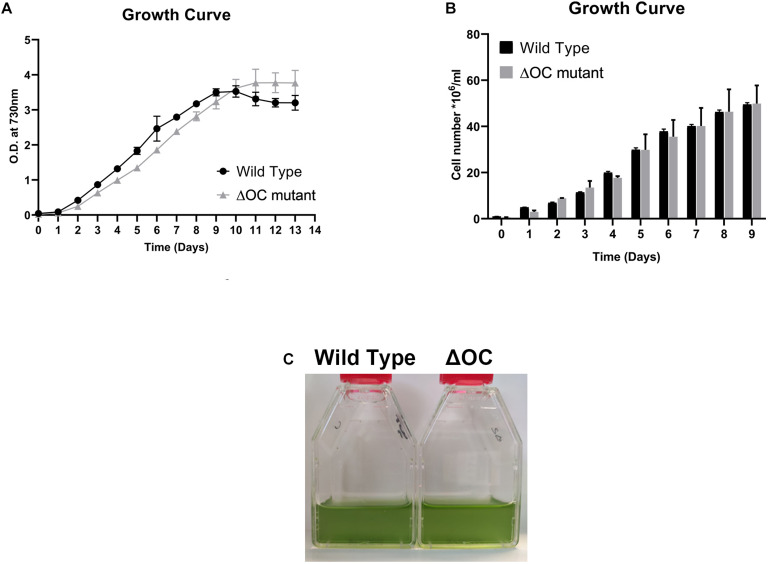
Growth of the *Synechocystis* 6803 wild type (WT) and ΔOC mutant. **(A)** Growth curve of *Synechocystis* 6803 wild type and ΔOC mutant strains monitored during 13 days by optical density (OD) measurements at 730 nm. Values are means ± SEM, *n* = 3. This experiment was repeated three times with similar results. **(B)** Quantification of the cell number of *Synechocystis* 6803 wild type and ΔOC mutant strains during growth for 9 days at control conditions. Values are means ± SEM, *n* = 3. This experiment was repeated three times with similar results. **(C)** Photos of representative wild type and ΔOC mutant strains at day 3 of growth at control conditions.

*In silico* analysis revealed the presence of a single pseudo-orthocaspase (SyOC) gene (*sll0148*) in *Synechocystis* sp. PCC 6803 containing the residues tyrosine (Y) –glycine (G) in the active site of the p20 domain instead of the conserved H-C dyad (Klemencic et al., 2019). To assess the function of the *Synechocystis* sp. PCC 6803 orthocaspase, mutants depleted of this pseudo-enzyme were constructed (Supplementary Figure 1), and their growth was compared to the WT cells (Figure 1A). At normal growth conditions no difference was observed in growth based on either OD_730_ (Figure 1A) or cell number (Figure 1B), or in the color of the culture (Figure 1C).

**FIGURE 2:**
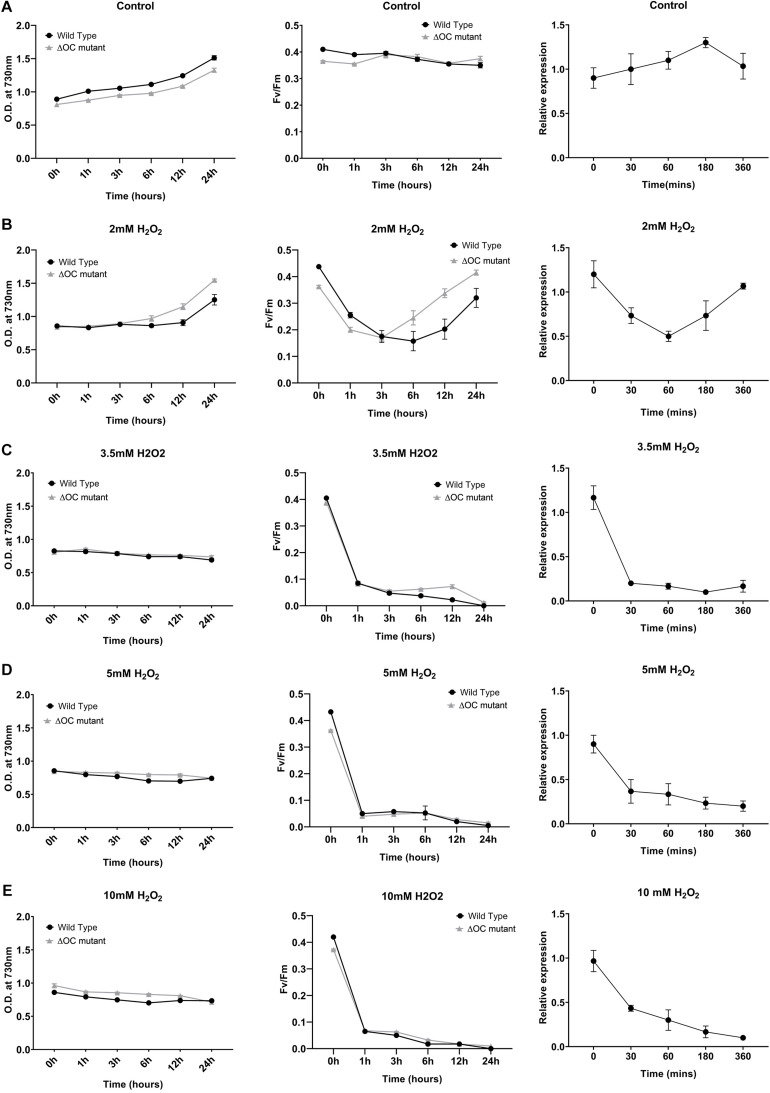
Growth of *Synechocystis* 6803 wild type and ΔOC mutant strains is impaired in the presence of hydrogen peroxide. *Synechocystis* 6803 wild type and ΔOC mutant strains in their exponential growth phase (OD ∼0.8) were exposed to different concentrations of H_2_O_2_ (**B:** 2, **C:** 3.5, **D:** 5, or **E:** 10 mM); non-treated cells were used as control **(A)**. Bacterial growth as OD_730_ (left panel) and quantum yield (Fv/Fm) (middle panel) was measured after 0, 1, 3, 6, 12, and 24 h of treatment. Right panel: Relative expression of *Sy*OC in WT exposed to H_2_O_2_ (2, 3.5, 5, or 10 mM) determined by real-time PCR. Relative expression to housekeeping genes *rnpB* and *16s* was determined at five different time points (0, 30, 60, 180, and 360 min) and plotted relative to 0 h (control condition). Data points are means (± SEM) calculated from three biological and three technical replicates and represent two independent experiments. Asterisk indicate a significant difference using ANOVA test *post-hoc* Fisher LSD (α = 0.05).

To investigate the putative role of SyOC during oxidative stress, we treated WT and ΔOC mutant cells with H_2_O_2_ at different concentrations (0, 2, 3.5, 5, or 10 mM, Figure 2, left panel). Samples were taken at the beginning of the experiment (*t* = 0) and after 1, 3, 6, 12, and 24 h of growth in the absence (Figure 2A) or presence (Figures 2B–E) of H_2_O_2_. As oxidative stress can inhibit the photosynthetic machinery (Nishiyama et al., 2001), we also monitored the photochemical efficiency during H_2_O_2_ treatment (Figure 2, middle panel). In the absence of H_2_O_2_, growth and photochemical quantum yield did not differ between WT and ΔOC (Figure 2A, middle panel); F_*V*_/F_*M*_ values around 0.4 are considered normal in cyanobacteria (Schuurmans et al., 2015). The growth rate (OD_730_, Figure 2, left panel) of WT and ΔOC cells was affected already at low H_2_O_2_ concentrations; however, while cells were able to recover in the presence of 2 mM H_2_O_2_ (Figure 2B, left panel), the growth stagnated at 3.5, 5, and 10 mM H_2_O_2_ (Figures 2C–E, left panel). The adaptation of *Synechocystis* sp. PCC 6803 to 2 mM H_2_O_2_ (Figure 2B, middle panel) also became obvious measuring the photochemical yield. During the first 3 h of growth in the presence of 2 mM H_2_O_2_, F_*V*_/F_*M*_ values dropped to about 0.2, but then the cells recovered to the normal values. Remarkably, the ΔOC mutant showed higher tolerance to 2 mM H_2_O_2_ than WT (Figure 2B, middle panel). While WT recovered only after 6 h of growth in 2 mM H_2_O_2_, ΔOC already recovered after 3 h and therefore seemed to be more tolerant to H_2_O_2_. Nevertheless, the growth rate and the photochemical yield were considerably affected after exposure to 3.5, 5, and 10 mM H_2_O_2_, and recovery was not observed within the experiment’s time frame (Figures 2C–E, middle panel).

To link the expression of the *syOC* gene to the observed H_2_O_2_ tolerance, RT-qPCR analysis was performed on WT cells grown in the presence of H_2_O_2_ as described above. Samples were collected at the beginning of the experiment (*t* = 0) and after 0.5, 1, 3, and 6 h of growth in the absence (Figure 2A, right panel) or presence of H_2_O_2_ (Figures 2B–E, right panel). While in control conditions *syOC* was continuously expressed, in the presence of H_2_O_2_ (independent of the concentration) the orthocaspase gene was down-regulated already after 30 min (Figures 2B–E, right panel). Interestingly, in the presence of 2 mM H_2_O_2_ expression of *syOC* decreased during the first hour, but later increased again to control level (Figure 2B, right panel). SyOC therefore might be important for normal growth at physiological conditions, but is redundant—or even counteractive—for adaptation to oxidative stress. A comprehensive study of this pseudo-orthocaspase gene of *Synechocystis* sp. PCC 6803 subjected to a broad range of stresses will be important in the future to clarify its expression patterns and activity.

### Depletion of *syOC* Increases the Survival Rate During Oxidative Stress

**FIGURE 3:**
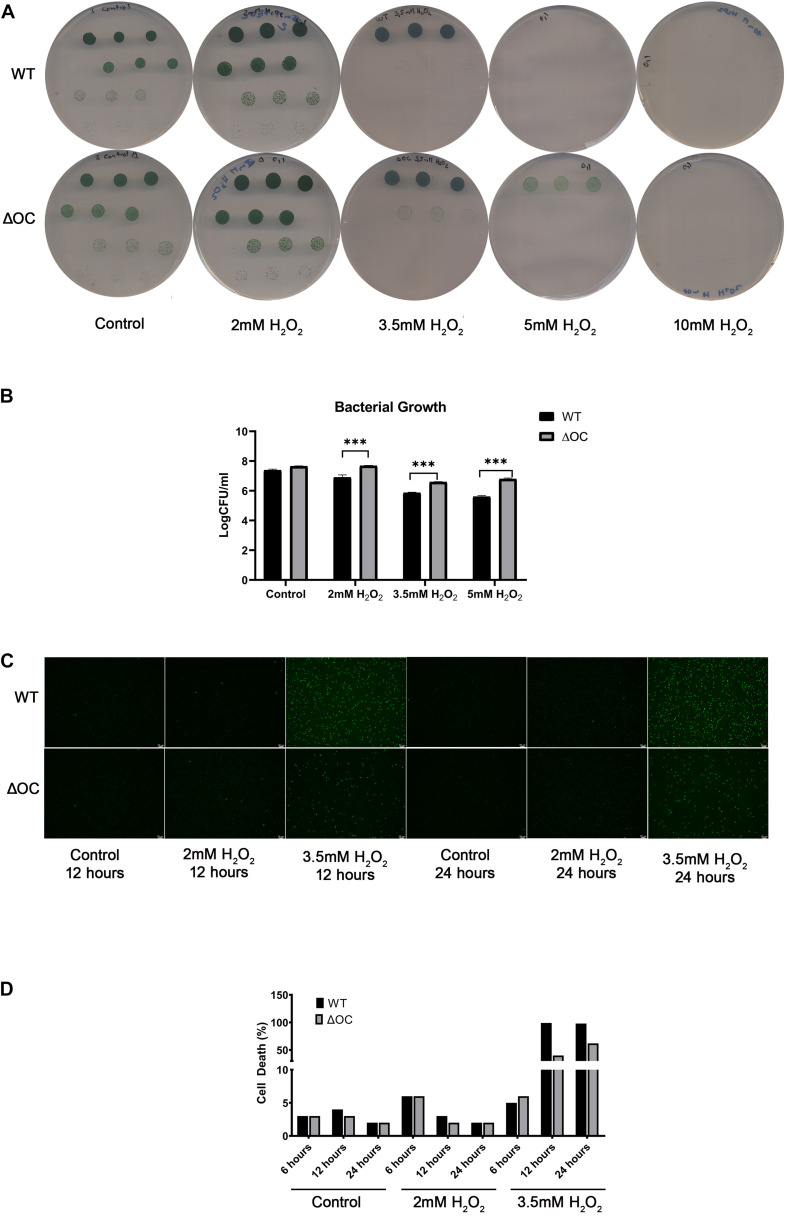
ΔOC survives growth in higher concentrations of hydrogen peroxide compared to the wild type. Photos **(A)** and evaluation **(B)** of drop dilutions (ranging from 10^–1^ to 10^–4^ fold) of WT and ΔOC *Synechocystis* strains using BG-11 plates in the absence or presence of different concentrations of H_2_O_2_. This experiment was repeated three times with similar results. Logarithmic values of CFU/ml are means (±SEM) calculated from three biological and three technical replicates. Photos **(C)** and quantification **(D)** of the cell death assay after growth in the presence of 2 or 3.5 mM H_2_O_2_ for 24 h. Dead cells (Sytox green-positive) were counted under a microscope. The experiment is representative of three independent replicates. Asterisks indicate statistical difference by *t*-test (α = 0.05).

To investigate a possible role of *syOC* in ROS-induced cell death, we performed a bacterial growth assay on BG-11 plates in the absence or presence of 2, 3.5, 5, or 10 mM H_2_O_2_. Cell suspension with an OD_730_ = 1 was diluted to decreasing concentrations ranging from 10^–1^ to 10^–4^, applied on the plates and grown for 4 days. Quantifying the number of colony forming units of wild type and ΔOC confirmed our previous results that high concentrations of H_2_O_2_ are lethal for *Synechocystis* sp. PCC 6803, as no colonies were observed even in highly concentrated starting droplets in the presence of 10 mM H_2_O_2_ (Figure 3A). WT cells were neither able to grow in the presence of 5 mM H_2_O_2_ and only in highest starting concentrations in the presence of 3.5 mM H_2_O_2_. However, the ΔOC mutant was more tolerant to H_2_O_2_; cells survived in the presence of 3.5 and even in the presence of 5 mM H_2_O_2_ (Figure 3A). We quantified the colony forming units at day 4 (Figure 3B). While growth of WT decreased already at an H_2_O_2_ concentration of 2 mM, the ΔOC mutant was not affected at this concentration (statistically significant, *t*-test, α = 0.05). Also at H_2_O_2_ concentrations of 3.5 and 5 mM the stress tolerance of the mutant was higher than that of WT, even though the growth rate decreased for both strains.

To determine the viability of cells under oxidative stress a fluorescence assay (Zheng et al., 2013) was performed in liquid media. The number of dead cells was investigated after 12 and 24 h in the presence of 2 or 3.5 mM H_2_O_2_ (Figures 3C,D). While after 12 h the number of dead WT cells in the presence of 2 mM H_2_O_2_ was higher than the number of dead ΔOC cells, after 24 h the number was similar in both strains (Figure 3D), supporting our previous results (Figure 2). In the presence of 3.5 mM H_2_O_2_, all WT cells (100%) were dead after 12 h, while 50% of the ΔOC cells still were alive even after 24 h (Figure 3D). The pseudo-orthocaspase therefore indeed might function in stress-perception in *Synechocystis* sp. PCC 6803.

### Identification of Differentially Expressed Proteins Under Oxidative Stress

**FIGURE 4:**
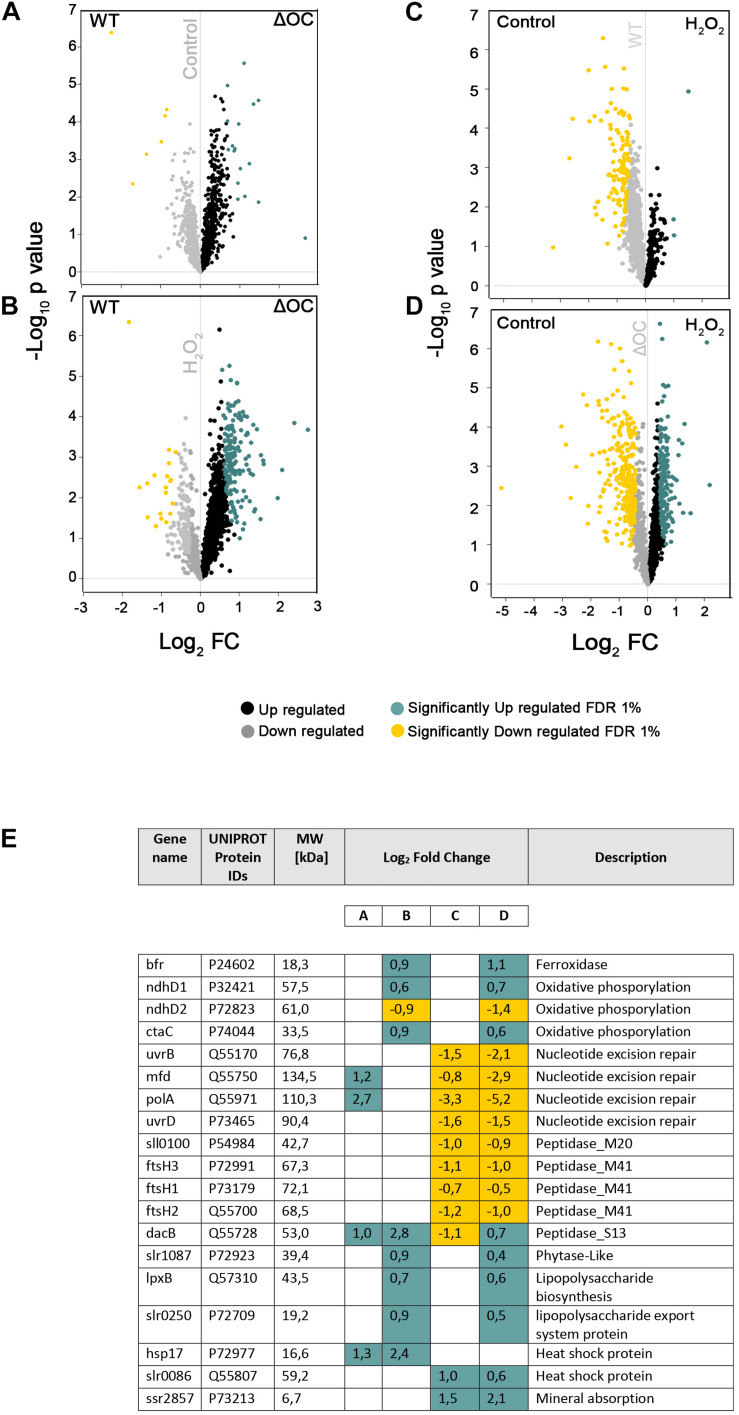
Proteomic changes in WT and ΔOC in response to H_2_O_2_. Volcano plots were constructed using Perseus. Logarithm of *p*-values plotted against the logarithm of fold change (FC) of the conditions indicated in each graph. **(A)** WT vs. ΔOC in control conditions; **(B)** WT vs. ΔOC exposed to H_2_O_2_ stress; **(C)** effect of H_2_O_2_ stress on WT; **(D)** effect of H_2_O_2_ stress on ΔOC. Up- or down-regulated proteins are shown in black and gray, respectively; significantly up- or down-regulated proteins in blue and yellow, respectively. **(E)** Significantly differentially regulated proteins involved in nucleotide excision repair, oxidative, lipopolysaccharide (biosynthesis and export), and stress-related processes (Heat shock and peptidases). Names as annotated in the UniProtKB database. Letters A–D correspond to the volcano plots **(A–D)**.

To address whether the absence of *syOC* had an impact on the proteome of *Synechocystis* sp. PCC 6803, proteins of four independent biological replicates of WT and the ΔOC mutant strains, grown in the presence or absence of 3.5 mM H_2_O_2_ for 1 h, were purified and identified by mass spectrometry. In total, 2,288 proteins across all tested conditions were detected. After filtering non-robust detections, 1,634 proteins remained. Significant changes in active protein abundance were detected using a two-sample test (see “Materials and Methods” section) and are summarized in Supplementary Tables 3, 4. Comparing the ΔOC mutant and WT grown at control condition, 17 proteins were upregulated in ΔOC (Supplementary Table 3). Among these proteins, two DNA binding proteins PolA and Mfd (Q55971 and Q55750, respectively) were observed, predicted to be involved in nucleotide excision repair, and a serine peptidase DacB (Q55728), which is involved in peptidoglycan biosynthesis according to the KEGG database (Figures 4A,E).

The differential analysis of ΔOC and WT exposed to H_2_O_2_ treatment (Figure 4B) identified 166 up-regulated and 19 down-regulated proteins (given in Supplementary Table 3). Among the listed proteins differing in concentration (Figure 4E and Supplementary Table 4), only two proteins (Q55807 and P73213) could be identified that were significantly upregulated under H_2_O_2_ stress in both strains, suggesting their importance in the process. Q55807 (*slr0086*) encodes a heat shock protein and P73213 (*ssr2857*) a protein involved in mineral absorption. While FtsH proteins were suggested to be essential for acclimation and starvation (Huang et al., 2019), in response to H_2_O_2_ stress FtsH proteins were down-regulated in WT and the mutant.

Volcano plots demonstrating changes in the proteome within one genotype (either WT or ΔOC) under the influence of H_2_O_2_ are shown in Figures 4C,D. H_2_O_2_ induced significant down-regulation of 128 proteins in WT and 290 in the ΔOC mutant strains (Figures 4C,D); among these, 96 proteins were detected in both genotypes and therefore seem to be specific for the response to H_2_O_2_ (Supplementary Table 3). SyOC was down-regulated in WT exposed to H_2_O_2_ (Supplementary Figure 2). Furthermore, 174 proteins were significantly up-regulated in ΔOC after exposure to H_2_O_2_ (Figure 4D), whereas only three proteins were up-regulated in WT comparing two groups (Figure 4C and Supplementary Table 3).

### Context-Specific Protein Networks Are Genotype-Dependent but Enlarged by Stress

**FIGURE 5:**
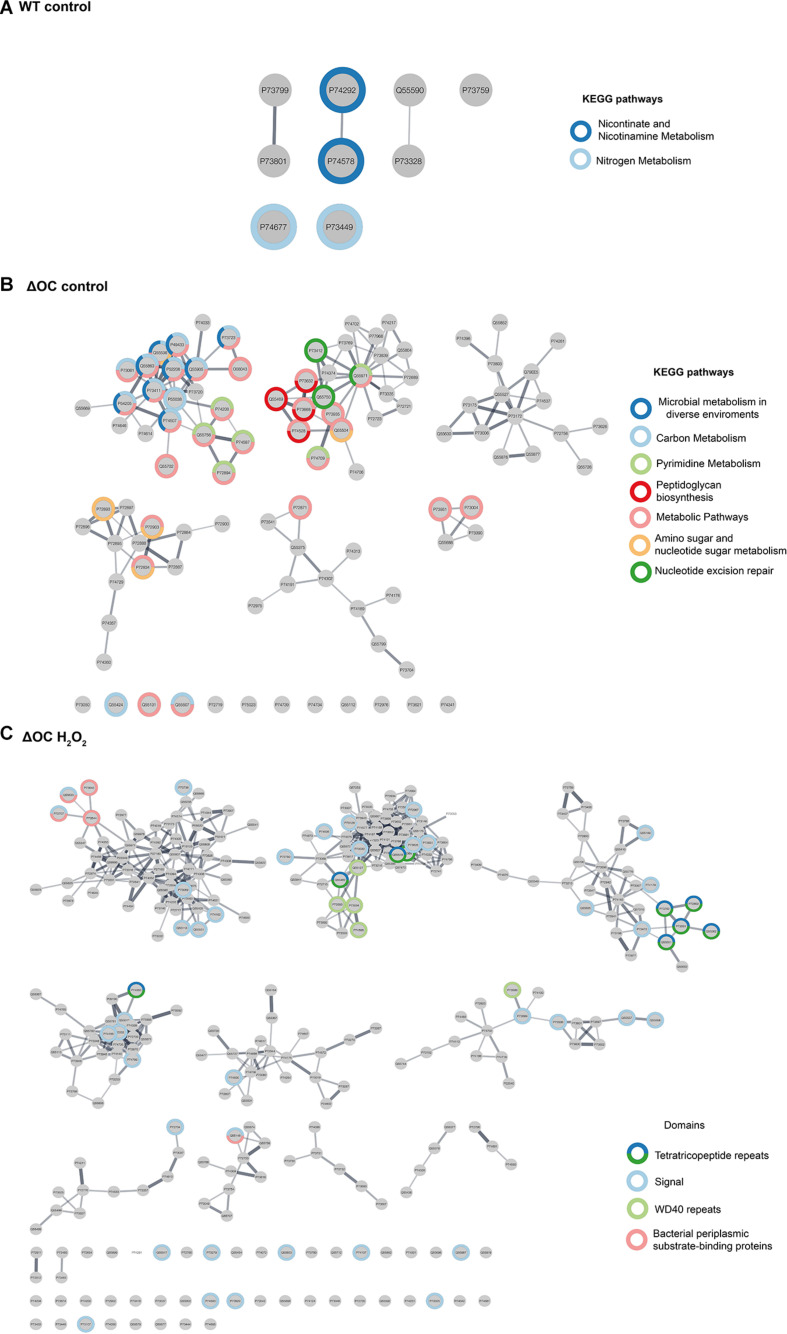
Strain-specific changes in protein interactions. Interaction networks for WT **(A)** and ΔOC **(B,C)**, protein- and KEGG pathways were annotated using the STRING databases and highlighted with different colors.

To understand how oxidative stress affects the interactome of the two strains, we generated four interaction networks (Hawaii plot) using a Perseus software, one per each experimental condition. Each protein, which was found to be upregulated at one condition, is represented by a node, and interactions are represented as edges (Figure 5). Within the networks, KEGG pathways, as well as pathways directly or indirectly related to stress responses, were highlighted. The resulting networks were composed of 9, 100, and 298 nodes (for WT control, ΔOC control and ΔOC H_2_O_2_, respectively) and 3, 152, and 701 edges, correspondingly. An interactome for WT exposed to H_2_O_2_ is not shown, as only one protein was significantly up-regulated in this analysis comparing all data; also in WT grown under control conditions very few proteins were up-regulated, which mainly function in nitrogen- and nicotinamine metabolism (Figure 5A). Community-based clustering revealed that in ΔOC when grown at control conditions proteins with a function in nucleotide excision repair as well as peptidoglycan pathways were up-regulated (Figure 5B), while growth in the presence of H_2_O_2_ induced proteins containing signal peptides, tetratricopeptide repeat (TPR) and beta-transducin repeat (WD40) motifs (Figure 5C). TPR motifs have been shown to participate in protein-protein interaction (Zeytuni and Zarivach, 2012). In general, network comparison indicated the ΔOC mutant strain to display a multilayer response to oxidative stress (Figure 5).

## Discussion

Mechanisms for handling environmental stress are crucial for all organisms, moreover for single cell organisms. Cyanobacteria have evolved the extraordinary capability to quickly adapt to fluctuating and damaging environmental conditions such as high light, oxidative stress, ultraviolet irradiation or high temperatures; several of them affecting both DNA and proteins (Latifi et al., 2009). They have developed numerous strategies such as activation or suppression of genes, and/or toxin-antitoxin (TA) systems (Hirani et al., 2001; Suzuki et al., 2004; Van Melderen and Saavedra De Bast, 2009; Coussens and Daines, 2016). Prokaryotic regulated cell death so far has not been considered to be an important mechanism in response to these environmental stimuli. RCD in unicellular organisms is a multi-layered and sophisticated process, combining structural (stacking of thylakoid membranes and reorganization of the chloroplast) (Durand et al., 2016) and molecular mechanisms (Engelberg-Kulka et al., 2006; Bayles, 2014), of which still very little is known.

Pseudo-enzymes are thought to arise after duplication of genes encoding the active enzyme and evolved to perform new cellular functions, for which the catalytic domain is not needed (Pils and Schultz, 2004; Murphy et al., 2017). However, in the majority of unicellular cyanobacteria, the only orthocaspase encoded in the genome is a pseudo-variant (Klemencic and Funk, 2018). Their evolutionary origin therefore remains unclear. Pseudo-orthocaspases are highly abundant in the oxygenic photosynthesis performing cyanobacteria.

In this study, we have characterized the pseudo-orthocaspase of *Synechocystis* sp. PCC 6803 (SyOC) with the aim to determine its possible role in cell surveillance. Even though the pseudo-enzyme is expressed at constant levels in WT grown under normal conditions, cells depleted of SyOC were not affected in growth rate and a phenotype different to WT was not obvious. Our data obtained in the proteomic analysis suggest that only very few proteins were regulated differentially in WT and ΔOC grown at normal conditions, the transcription-repair coupling factor (MFD) and the DNA polymerase I (PolA) were up-regulated in ΔOC (Figure 4A). During DNA repair Mfd was found to remove the RNA polymerase, deliver the repair enzyme to the lesion, and thereby facilitating the recruitment of DNA repair proteins (Park et al., 2002; Selby, 2017).

After exposure to oxidative stress, the amount of SyOC in WT was down-regulated at the gene (Figure 2) and protein (Supplementary Figure 2) level. Still, the main difference between ΔOC and WT strain was an increased tolerance to H_2_O_2_, visible by faster adaptation to oxidative stress (Figure 2B), increased colony number formation (CFU) and lower number of dead cells (Figure 3) during growth in the presence of H_2_O_2_. In the presence of H_2_O_2_, several proteins were significantly up-regulated in ΔOC (Figures 4D, 5C). Interestingly, numerous of these proteins contain TRP and WD40 motifs, which have been found to be involved in protein-protein interactions and oligomerization of proteins related to cell death in plants and animals (Tavernarakis et al., 1999; Weber and Vincenz, 2001; Stirnimann et al., 2010; Gehl and Sweetlove, 2014). After exposure to H_2_O_2_, concentrations of the membrane proteins GumB (73198) and PHB (P72754) were up-regulated in ΔOC (Figure 4D). GumB has been shown to play a role in polysaccharide export in bacteria (Galvan et al., 2013) and PHB belongs to the band-7 protein family proteins. The exact function of the band-7 domain is still unknown in most organisms, but proteins with this domain have been demonstrated to bind to lipids and to assemble into membrane-bound oligomers (Tavernarakis et al., 1999). They are involved in creating areas of protein quality control and play a role in hypersensitive response in plants (Gehl and Sweetlove, 2014). Further, they seem to be involved in cell survival and apoptosis in animals (Peng et al., 2015). Our screen revealed several other unannotated/uncharacterized proteins implicated in response to abiotic stress, opening new avenues for molecular dissection of the pseudo orthocaspases.

*Synechocystis* sp. PCC 6803 has been extensively studied after exposure to various stresses; however, while sensor histidine kinases (Hik), small CAB-like proteins (S), or proteases like HtrA, Clp, or FtsH are known regulators of protein homeostasis in photosynthetic prokaryotes (Stanne et al., 2007; Tryggvesson et al., 2012; Cheregi et al., 2016; Tibiletti et al., 2018; Huang et al., 2019), pseudo-enzymes, particularly SyOC, have so far escaped detection. Regulated proteolytic activity is an essential mechanism of signal transduction used by peptidases to cleavage substrates and response to external stimuli (Wettstadt and Llamas, 2020). Nevertheless, our results highlight the relevance of pseudo-enzymes in maintaining internal homeostasis. The molecular involvement of SyOC in cell survival during normal growth conditions (since it is constitutively expressed) and H_2_O_2_ tolerance remains to be clarified.

The expression of the orthocaspases of the marine cyanobacterium *Trichodesmium* has previously been shown to be upregulated during Fe-limitation or during a combination of Fe- and light stress (oxidative stress) (Spungin et al., 2019). Unfortunately, due to their mutations in the catalytic dyad, the pseudo-orthocaspases TeMC11 and TeMC12 have not been analyzed in this study (Spungin et al., 2019). We found SyOC constantly expressed during normal conditions in continuous light, however, during growth in the presence of H_2_O_2_ expression was down-regulated during the adaptation process on gene and protein level (Figure 2 and Supplementary Figure 2). Therefore, lack of SyOC seems to be essential to induce tolerance to oxidative stress (as in ΔOC). In that sense, SyOC could be involved in a pro-death pathway during oxidative stress. Further analyses are needed to confirm this hypothesis. Nevertheless, the presence or absence of the proteolytic domain in orthocaspases might lead to diverging evolutionary pathways in cyanobacteria.

In summary, for the first time, we highlighted the importance of a pseudo-orthocaspase and now propose SyOC of *Synechocystis* sp. PCC 6803 to play a role in response to oxidative stress.

## Data Availability Statement

The data presented in the study are deposited in the ProteomeXchange Consortium via the PRIDE (Vizcaino et al., 2016) partner repository with the dataset identifier PXD022955.

## Author Contributions

SLA and MK performed the formal analysis, generated, and characterized the mutant strain. FV and MA performed mass spectrometry experiments. SLA, FV, and MA analyzed the mass spectrometry data. CF conceived and supervised the project, conceptualized the project, and was responsible for funding acquisition. SLA and CF wrote the manuscript with contributions from all authors.

## Conflict of Interest

The authors declare that the research was conducted in the absence of any commercial or financial relationships that could be construed as a potential conflict of interest.

## Footnotes

1 http://genome.annotation.jp/cyanobase/Synechocystis

2 https://www.ncbi.nlm.nih.gov/tools/primer-blast/

3 https://www.ebi.ac.uk/pride/archive/
