# Supplementary material for: The Role of Pseudo-Orthocaspase (SyOC) of Synechocystis sp. PCC 6803 in Attenuating the Effect of Oxidative Stress
Source: Front Microbiol. 2021 Feb 4;12:634366. doi: 10.3389/fmicb.2021.634366 (PMC7889975; doi:10.3389/fmicb.2021.634366)
Supplement: Supplementary file 1 [file Data_Sheet_1.docx]

**The proteolytically inactive orthocaspase of *Synechocystis sp.* PCC 6803 plays an important role in oxidative stress-induced cell death response**

Saul Lema^1^, Marina Klemencic^1#^, Franziska Völlmy^2,3^, Maarten Altelaar^2,3^ and Christiane Funk^1^*

^1^Dept of Chemistry, Umeå University, Umeå, Sweden.

^2^Biomolecular Mass Spectrometry and Proteomics, Bijvoet Center for Biomolecular Research and Utrecht Institute for Pharmaceutical Sciences, University of Utrecht, Utrecht, The Netherlands

^3^Netherlands Proteomics Centre, Utrecht, The Netherlands

^#^current address: Faculty of Chemistry and Chemical Technology, University of Ljubljana, Ljubljana, Slovenia

*Corresponding Author

**Supplementary Data**

**
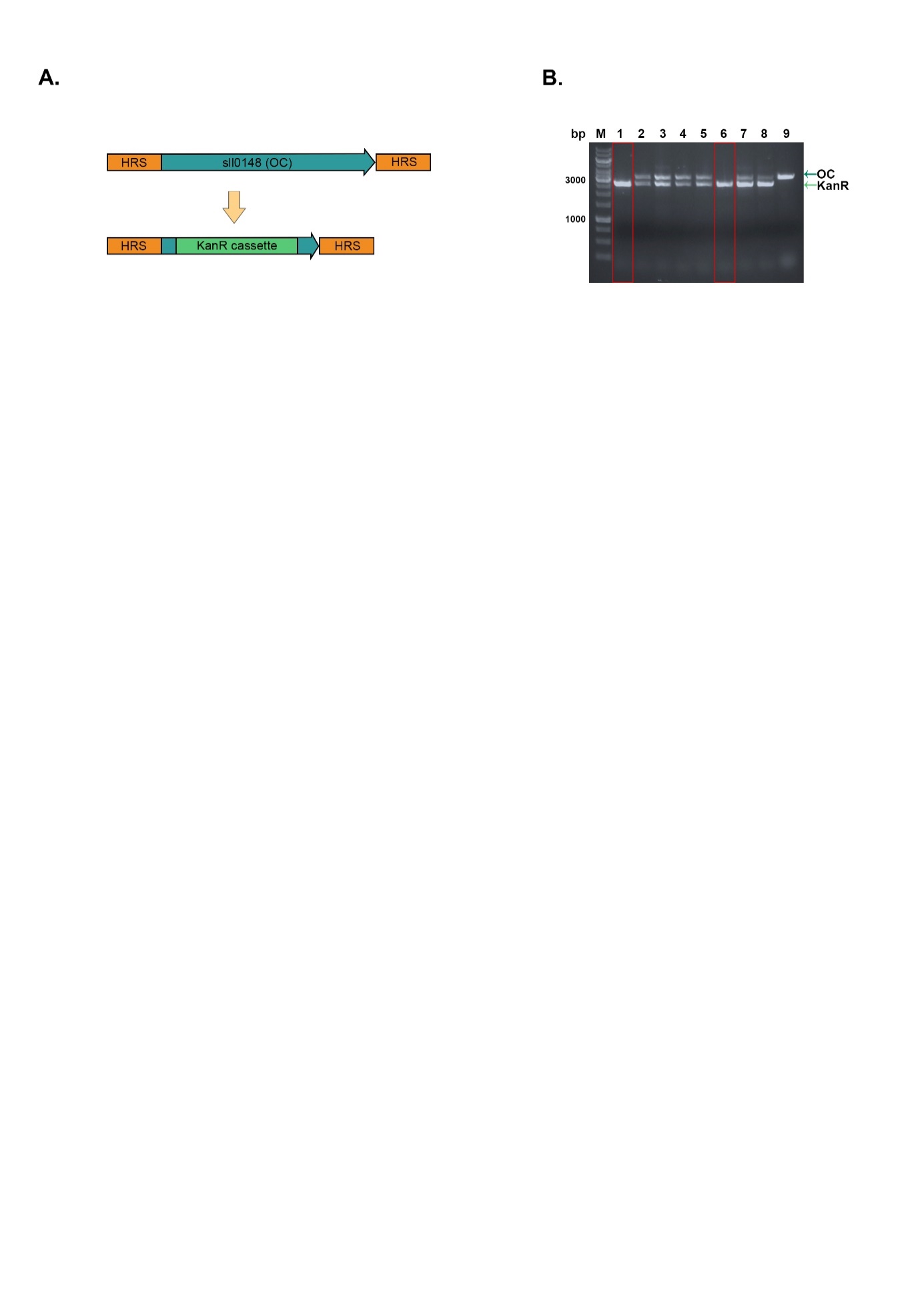
Supplementary Figure 1. Schematic representation of ∆OC mutant generation.** (A) Schematic representation of *sll0148* and its disruption by a kanamycin-resistance cassette. (B) PCR identification of suitable mutants after segregation. Red boxes represent positive candidates selected for further analysis.


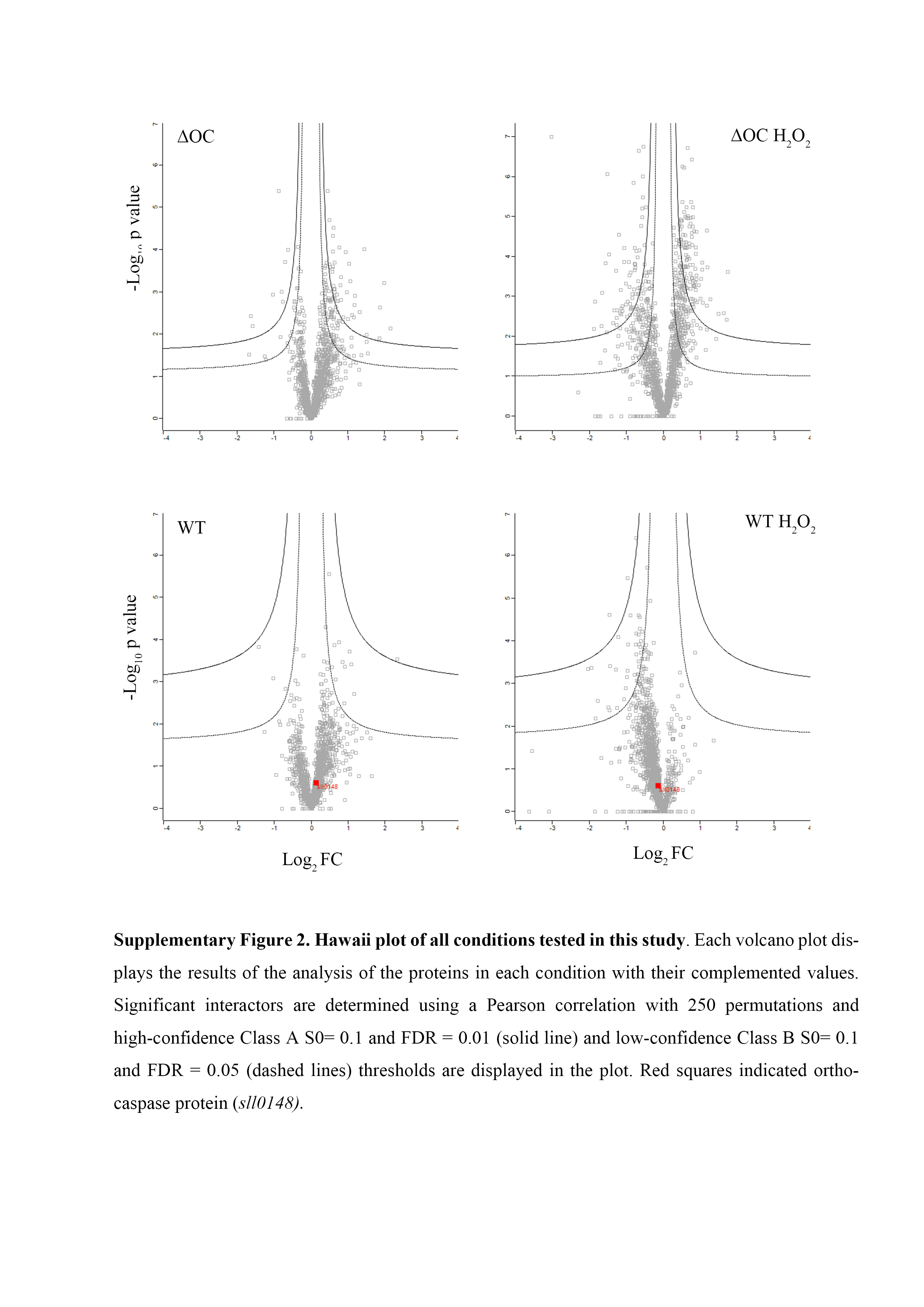


**Supplementary Figure 2. Hawaii plot displaying proteomic changes in the conditions tested**. Volcano plots comparing protein abundance in the two genotypes (WT or ∆OC) at each growth condition (control or presence of 3.5 mM H_2_O_2_ for 1 hour). Significant interactors are determined using a Pearson correlation with 250 permutations and high-confidence Class A S_0_= 0.1 and FDR = 0.01 (solid line) and low-confidence Class B S_0_= 0.1 and FDR = 0.05 (dashed lines) thresholds are displayed in the plot.

| **Name of Primer** | **Sequence** | **Description** |
| --- | --- | --- |
| deltaSyOC_Eco_F | ATCTGATGAATTCCCCGGTTGCGGACAAC | Mutant construction |
| deltaSyOC_Eco_R | ATCAGATCTGCAGTTCTTTGGTCTATTGGCAGACTCG | Mutant construction |
| Kan_Apa_F | TTCACGTGGGCCCAGGAAACAGCTATGACCATGATTACG | Mutant construction |
| Kan_SmaI_R | CTTCACGTCCCGGGGTTGTAAAACGACGGCCAGTG | Mutant construction |
| SyOC_F | TAGGCAATTGCTGGGGGAAG | Real time PCR |
| SyOC_R | AATTTCCAGGGCTGACAGGG | Real time PCR |
| Sy16S_F | CTTCCGGTACGGCTACCTTG | Real time PCR |
| Sy16S_R | CGCCCGAAGTCGTTACTCTA | Real time PCR |
| SyrnpB_F | TGATTGGTGGAACCGCTTGA | Real time PCR |
| SyrnpB_R | GTAAGCCGGGTTCTGTTCCA | Real time PCR |

**Supplementary Table 1. List of all primers used in this study**

**Supplementary Table 2. log_2_ LFQ intensities of the proteomic data**. Detection for every protein and sample analyzed; Peptide counts (all, razor + unique, and unique); Sequence coverage % (all, razor + unique, unique); Molecular weight of the protein; Score values, Spectral counts; Protein and Majority Protein IDs (UniProtKB); Gene name; Uniprot Names; Protein name; PFAM domains; KEGG name and Interpro name.

*Given as separate Excel file*

**Supplementary Table 3**. **Differentially expressed proteins between the two genotypes and two growth conditions.** (A) Control condition: ∆OC vs WT. (B) Growth in the presence of H_2_O_2_: ∆OC vs WT (C) Wild type: presence of H_2_O_2_ vs control growth conditions (D) ∆OC mutant: presence of H_2_O_2_ vs control growth conditions. Up-regulated proteins are marked in dark teal, down-regulated proteins in gold.

*Given as separate Excel file*

**Supplementary table 4.** **List of differentially expressed proteins identified in more than one comparison.** log2 LFQ intensities of protein detections; GOBP name, GOMF name, GOCC name, KEGG name and PFAM domains. Up-regulated proteins are marked in dark teal, down-regulated proteins in gold. (A) WT versus ∆OC in control conditions; (B) WT versus ∆OC exposed to H_2_O_2_ stress; (C) effect of H_2_O_2_ stress on WT; (D) effect of H_2_O_2_ stress on ∆OC.

*Given as separate Excel file*
